# Canine Antibodies against Salivary Recombinant Proteins of *Phlebotomus perniciosus*: A Longitudinal Study in an Endemic Focus of Canine Leishmaniasis

**DOI:** 10.1371/journal.pntd.0003855

**Published:** 2015-06-25

**Authors:** Tatiana Kostalova, Tereza Lestinova, Petra Sumova, Michaela Vlkova, Iva Rohousova, Eduardo Berriatua, Gaetano Oliva, Eleonora Fiorentino, Aldo Scalone, Marina Gramiccia, Luigi Gradoni, Petr Volf

**Affiliations:** 1 Department of Parasitology, Faculty of Science, Charles University in Prague, Prague, Czech Republic; 2 Animal Health Department, Universidad de Murcia, Murcia, Spain; 3 Department of Veterinary Medicine and Animal Production, University Federico II, Naples, Italy; 4 Unit of Vector-borne Diseases and International Health, Istituto Superiore di Sanità, Rome, Italy; US Food and Drug Administration, UNITED STATES

## Abstract

**Background:**

Phlebotomine sand flies are vectors of *Leishmania* parasites. During blood feeding, sand flies deposit into the host skin immunogenic salivary proteins which elicit specific antibody responses. These anti-saliva antibodies enable an estimate of the host exposure to sand flies and, in leishmaniasis endemic areas, also the risk for *Leishmania* infections. However, the use of whole salivary gland homogenates as antigen has several limitations, and therefore, recombinant salivary proteins have been tested to replace them in antibody detection assays. In this study, we have used for the first time sand fly salivary recombinant proteins in a longitudinal field study on dogs.

**Methodology/Principal Findings:**

Sera from dogs naturally exposed to *P*. *perniciosus* bites over two consecutive transmission seasons in a site endemic for canine leishmaniasis (CanL) were tested at different time points by ELISA for the antibodies recognizing whole saliva, single salivary 43 kDa yellow-related recombinant protein (rSP03B), and a combination of two salivary recombinant proteins, 43 kDa yellow-related protein and 35.5 kDa apyrase (rSP01). Dogs were also tested for *Leishmania infantum* positivity by serology, culture, and PCR and the infection status was evaluated prospectively. We found a significant association between active CanL infection and the amount of anti-*P*. *perniciosus* saliva antibodies. Importantly, we detected a high correlation between IgG antibodies recognizing rSP03B protein and the whole salivary antigen. The kinetics of antibody response showed for both a whole saliva and rSP03B a similar pattern that was clearly related to the seasonal abundance of *P*. *perniciosus*.

**Conclusions:**

These results suggest that *P*. *perniciosus* rSP03B protein is a valid alternative to whole saliva and could be used in large-scale serological studies. This novel method could be a practical and economically-sound tool to detect the host exposure to sand fly bites in CanL endemic areas.

## Author Summary

Canine leishmaniasis (CanL) is a widespread zoonosis caused by protozoan parasite *Leishmania infantum*. CanL is endemic in more than 70 countries, including regions of southwestern Europe, where the main vector is *Phlebotomus perniciosus*. Saliva inoculated into the host during blood feeding provoke species-specific antibody response. They could be used as a serological marker of exposure to sand flies and, in leishmaniasis endemic areas, also as an indication of *Leishmania* infection risk. However, in case of large-scale serological studies, it is difficult to obtain appropriate amounts of crude salivary antigen from laboratory-reared sand flies. Therefore, we studied if specific salivary recombinant proteins could be suitable to replace whole saliva as an antigen for serological assays. Antibodies recognizing recombinant salivary protein in naturally bitten dogs were highly correlated with antibody response against whole saliva. Seasonal exposure of dogs to sand flies led to antibody response fluctuations related to the period of activity and abundance of vectors. In a context of high CanL endemicity, we found a significant association between progressive CanL infections and antibodies against sand fly saliva. Thus, salivary recombinant proteins could be used as suitable tool for investigations host exposure to sand flies in endemic settings.

## Introduction

Canine leishmaniasis (CanL), caused by protozoan parasite *Leishmania infantum*, is a systemic and potentially fatal disease [reviewed in [1, 2]]. It may affect any organ or body fluid [reviewed in [1]] and can manifest variable clinical signs [reviewed in [2, 3]]. However, the majority of infected dogs do not develop any clinical signs. Importantly, *L*. *infantum* is also a causative agent of human visceral leishmaniasis and both symptomatic and asymptomatic dogs have a crucial role in the epidemiology of this disease, serving as reservoirs [reviewed in [2]]. CanL is endemic in many regions of southern Europe and Latin America, however, climate changes and socioeconomic factors, particularly increased travelling of dogs between endemic and non-endemic areas, led to changes in the distribution of CanL in both continents [reviewed in [1, 2, 4]].

Diagnosis of CanL should be based on an integrated approach considering signalment, history, clinical findings, and results of basic laboratory analyses that detect the parasite or evaluate the immune response in the host [reviewed in [3]]. The commonly used diagnostic methods include direct detection of the parasite by culture or polymerase chain reaction (PCR) and indirect techniques for detection of antibodies against *Leishmania*, such as immunofluorescent antibody test (IFAT) and enzyme-linked immunosorbent assay (ELISA) [5–8]. However, there is still lack of practical methods for detection of the risk of *Leishmania* transmission according to the exposure of dogs to sand fly vectors.

Results from studies on human and canine antibody response against sand fly saliva revealed high immunogenicity of sand fly salivary proteins as well as high specificity of anti-saliva IgG [9–12]. Studies based on dynamics of IgG response in dogs experimentally exposed to the main *L*. *infantum* vectors, *Lutzomyia longipalpis* and *Phlebotomus perniciosus* [12, 13], suggest that monitoring canine antibody response to sand fly saliva could be a useful epidemiological tool in CanL foci. However, the use of whole sand fly saliva in such studies is limited by time-consuming salivary gland dissection and complicated by potential cross-reactivity with saliva from sand fly species with no role in *Leishmania* transmission [11] or from other blood sucking insects [14]. These problems could be overcome by using specific immunogenic sand fly salivary recombinant proteins [reviewed in [15]]. The use of recombinant proteins was already shown for *P*. *papatasi* [16, 17], *Lu*. *longipalpis* [18, 19] and *P*. *perniciosus* [20, 21]. Although these studies confirmed the advantages of salivary recombinant proteins, most of them were tested on small sets of samples and never in association with naturally transmitted *Leishmania* infections.

Previous studies on different hosts (dogs, foxes, and humans) bitten by *P*. *perniciosus* or *Lu*. *longipalpis* revealed high antigenicity of salivary yellow-related proteins and apyrases [12, 13, 19]. Apyrases are enzymes with potent anti-hemostatic activity hydrolyzing the platelet activator ADP [22]. The role of yellow-related proteins is less clear [23] but most probably they act as a histamin-binding molecules [24]. Recent work using a low number of sera from dogs experimentally exposed to *P*. *perniciosus* showed that recombinant forms of these proteins could be used as potential candidates for markers of canine exposure to sand flies [20]. Therefore, herein we used the recombinant 43 kDa yellow-related protein (rSP03B) from *P*. *perniciosus* and its combination with recombinant *P*. *perniciosus* 35.5 kDa apyrase (rSP01) to study the specific antibody response in a large number of dogs naturally exposed to *P*. *perniciosus* over two years in a focus endemic for *L*. *infantum*.

## Methods

### Ethical statement

The technical protocol for the investigation of natural canine *Leishmania* infections performed in the frame of experimental drug trials was approved by the Veterinary Board of the Italian Ministry of Health (authorization no.4051/P) following the European Directive 86/609/EEC, adopted by the Italian Government with the Law 116/1992.

### Experimental design and background

A longitudinal study including two sand fly seasons was performed on 56 Beagle dogs housed in the same open-air kennel sited in a rural municipality of the Naples province (Campania region, southern Italy) where both human visceral leishmaniasis and CanL are highly endemic. Here, *P*. *perniciosus* is the only *Leishmania* vector, whose activity period ranges from the end of May through late October [25]. *Leishmania infantum* infection rates in dissected sand flies were found to range from 2.8% to 6.2% in places not far from the kennel [26]. In cohorts of naïve dogs previously exposed in the same kennel, the annual incidence of CanL infection and clinical disease was reported to average about 40% and 20%, respectively [8]. The dogs included in our study were part of experimental trials of pharmacological products against CanL. The animals were born in a breeding facility sited in a non-endemic area of northern Europe, and were confirmed to be CanL-free at the time they were moved to the study site when they were about 6 months old. Once housed in the open-air kennel, the use of topical or environmental insecticides was avoided to allow natural exposure of dogs to sand fly bites in the warm season.

Antibodies against sand fly salivary proteins were analyzed retrospectively in dog sera taken for routine clinical examination and periodical CanL serology, and that were stored frozen. Available samples included sera obtained shortly before the dogs were transferred to the endemic area in July (first year), and they served as pre-immune sera (n = 56). Responses to salivary antigens were then analyzed on selected samples collected at four-time points in the first year [August (n = 53), September (n = 54), October (n = 34), December (n = 54)] and at five occasions during the second year [January (n = 56), March (n = 56), July (n = 56), August (n = 56), September (n = 54)]. As per established protocols [5], follow-up analyses for the detection and classification of natural CanL infections were performed at the beginning of the study in the first year in July (n = 56), twice in the second year [March (n = 56) and July (n = 56)] and once at the beginning of the third year [March (n = 53)].

### Analysis of *Leishmania* infection

Dogs were examined by serology, culture, and PCR. Detection of anti-*Leishmania* IgG antibodies was performed by an in-house IFAT assay using *L*. *infantum* promastigotes as antigen and following the protocol recommended by the Office International des Epizooties [27]. The cut-off dilution for *Leishmania* exposure was set at 1:40 (i.e. the upper part of the so-called “IFAT grey zone”) [3]. Bone-marrow aspirate material was examined by nested-PCR assay as previously described [28]. Lymph-node aspirate material was cultured from each popliteal node in Evans’ Modified Tobie’s medium and cultures were periodically examined for promastigotes growth during one month.

At each assessment, the dogs were classified as follows with regards to the infection status: i) “*Leishmania* negative”if found negative by all assays, ii)”*Leishmania* exposed”if tested positive by IFAT at low titers and negative by other tests, iii) having a”subpatent *Leishmania* infection” if bone-marrow PCR tested positive, IFAT was either negative or positive at low titers, and lymph node culture was negative, iv) having an”active *Leishmania* infection” if both bone-marrow PCR and lymph node culture were tested positive. In this infection stage, IFAT can be found negative or positive at low titer initially, but converts shortly to very elevated titers. Once established, active infections do not regress spontaneously towards negative or subpatent conditions and dogs invariably progress to clinical disease [8].

### Sand flies and salivary proteins

A colony of *P*. *perniciosus* was reared under standard conditions as described in [29]. Salivary glands, dissected from 4–6 day old female sand flies, were pooled in 20 mM Tris buffer with 150 mM NaCl and stored at -20°C. Recombinant salivary proteins from *P*. *perniciosus*, 35.5 kDa apyrase (rSP01, Genbank accn. DQ192490) and 43 kDa yellow-related protein (rSP03B, Genbank accn. DQ150622) were obtained from Apronex s.r.o. (Prague) as mentioned in [20]. The concentrations of these proteins were quantified by the Lowry method (Bio-Rad) following the manufacturer´s protocol.

### Detection of anti- *P*. *perniciosus* IgG

Anti-*P*. *perniciosus* IgG were measured by enzyme-linked immunosorbent assay (ELISA) as described in [12] with minor modifications. Briefly, microtiter plates were coated either with salivary gland homogenate (SGH) (0.2 salivary gland per well) or with rSP03B (5μg/ml) or with rSP03B+rSP01 (5μg/ml of each protein) in 20 mM carbonate-bicarbonate buffer (pH 9.5) overnight at 4°C. The plates were incubated with blocking solution, 6% (w/v) low fat dry milk in PBS with 0.05% Tween 20 (PBS-Tw). Canine sera were diluted 1:200 for SGH and 1:100 for recombinant proteins in 2% (w/v) low fat dry milk/PBS-Tw. Secondary antibodies (anti-dog IgG, Bethyl laboratories) were diluted 1:9000 in PBS-Tw. Absorbance was measured at 492 nm using a Tecan Infinite M200 microplate reader (Schoeller). Each serum was tested in duplicate and the experiment was repeated twice.

### Statistical analysis

Antibodies against sand fly saliva are reported as optical densities (OD) with subtracted blanks and multiplied by 100 for easier readability. In case of linear mixed models, ODs were log transformed (OD value+1).

Statistical analyses were carried out using R software (http://cran.r-project.org/). Repeatability defined as the degree of agreement between repeated OD measures on the same samples was estimated within and between plates by calculating the concordance correlation coefficient (CCC) that ranges from 0 (no concordance) to 1 (perfect concordance) [30].

Antibodies against sand fly saliva and *Leishmania* infection status frequency distributions were analysed over time. Proportions and medians between time points were compared using McNemar’s chi-square test for paired data and Wilcoxon signed rank sum test, respectively. Correlations were analysed using Spearman rank correlation test [31].

The “nlme” package [32] was then used to develop multilevel linear regression models to investigate the relationship between IgG anti-saliva antibodies as continuous dependent variable, and sampling months and *L*. *infantum* infection status (as described above) included as categorical predictor variables, taking into account the correlation between repeated measures of the same dogs over time. Two hierarchical levels were considered in the analysis with repeated measures within individual dogs as the level-1 units and individual dogs as the level-2 units. The random variation at the dog level was examined at both the intercept and at the slopes and significance was assessed testing the -2 log likelihood ratios between the model with and the model without the random effect [33]. The correlation between level 1 units was considered as having a compound-symmetry (CS) or autoregressive with a lag of 1 (AR-1) structure or as unstructured (UN) and Akaikes’s Information Criterion (AIC) was used to compare the goodness of fit of models with different correlation structures, selecting those with the smallest values of these statistics [33, 34]. Parameter estimates for fixed effects were estimated using restricted maximum likelihood estimation (REML) and significance was assessed using conditional t-tests and F-tests, and alpha was taken at the 5% (p<0.05) level for a two-tailed test.

## Results

### Dynamics of *L*. *infantum* infection status

**Fig 1:**
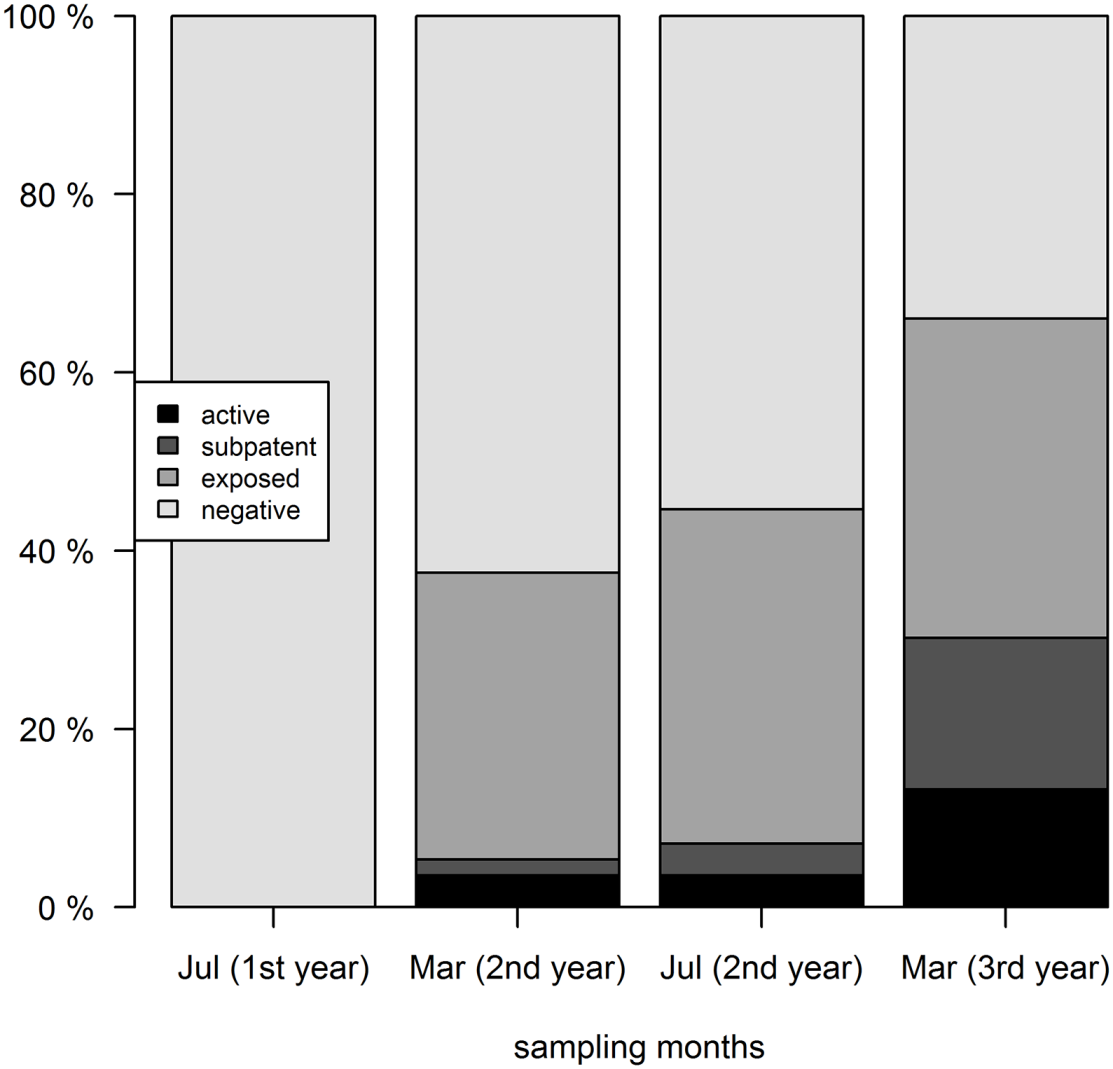
Dynamics of *L*. *infantum* infection statuses in dogs from a CanL endemic site in southern Italy. Dogs were screened for *L*. *infantum* infection on four occasions, 56 dogs in July first year, 56 dogs in March the second year, 56 dogs in July the second year and 53 dogs in March the third year.

The percentage of dogs classified as negative, *Leishmania* exposed, subpatently and actively infected is presented in Fig 1.

At the beginning of the study (July in the first year) all dogs were negative by all tests, thereafter, the proportion of *L*. *infantum* infected dogs increased throughout the trial (p<0.05) (Fig 1). In the following sampling, in March, 32% of dogs were classified as *Leishmania* exposed, while subpatent and active infections were found in 2% and 4% of dogs, respectively. The percentage of dogs in each category was similar 3 months later in July (Fig 1). In contrast, by the end of the study in March of the third year, the percentage of subpatently and actively infected dogs sharply increased to 17% and 13%, respectively. By this time, 36% of dogs were classified as *Leishmania* exposed and 34% as negative (p<0.05) (Fig 1).

### Distribution and dynamics of antibody response against salivary proteins using SGH, rSP03B, and rSP03B+rSP01 as antigens

**Fig 2:**
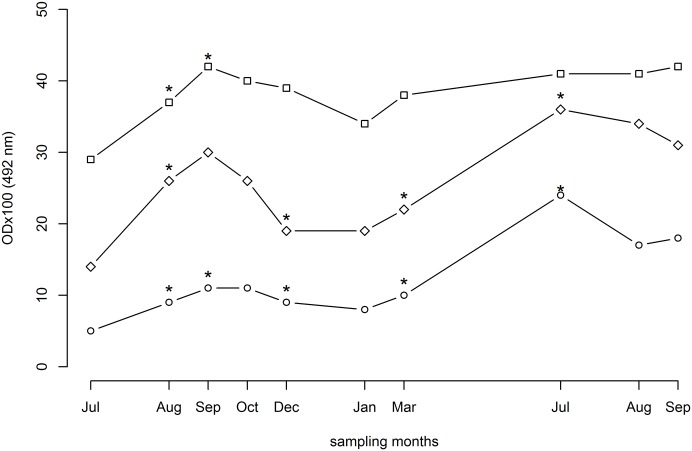
Dynamics of IgG antibody response against sand fly salivary proteins in dogs naturally exposed to *P*. *perniciosus* over two years in endemic foci. Canine sera were tested by ELISA for the antibodies recognizing SGH (open circle), rSP03B protein (open diamond) and combination of rSP03B+rSP01 proteins (open square). Data are presented as median values for each sampling month. Asterisk represents significant change in the median compared to previous sampling. ODx100 = optical density multiplied by 100.

**Fig 3:**
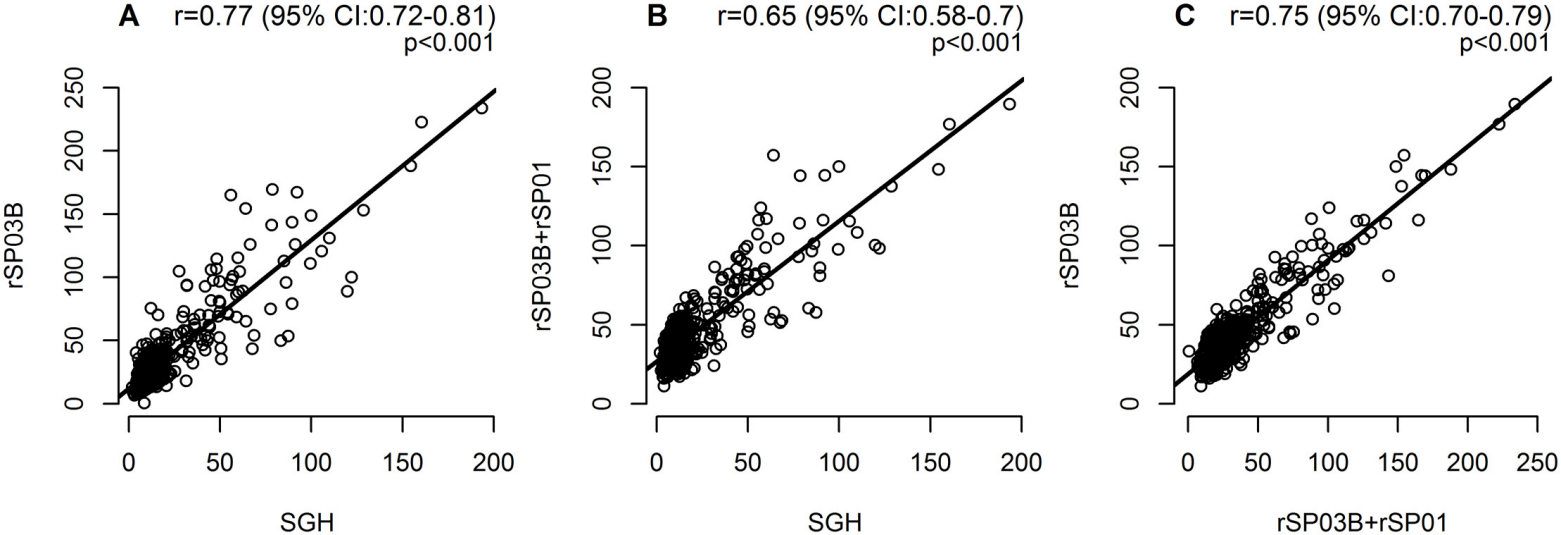
Correlations between IgG antibodies recognizing SGH and recombinant salivary proteins in dogs naturally exposed to sand flies. Correlation between SGH and rSP03B protein (A), between SGH and combination of rSP03B+rSP01proteins (B) and between rSP03B protein and combination of rSP03B+rSP01proteins (C) was performed using Spearman rank correlation. OD values were multiplied by 100. r = correlation index, CI = confidence interval.

The overall median values of ELISA ODx100 using SGH, rSP03B, and rSP03B+rSP01 antigens were 10 (range: 2–194), 24 (1–234) and 37 (11–189), respectively (p<0.05). However, the median increased significantly with time, following a similar pattern for all three antigens tested, most significantly for SGH and rSP03B (Fig 2). For example, the median ODx100 for SGH was 5 at the baseline in July of the first year, increased significantly to 11 through October, decreased thereafter to 8 in January, increased again up to a peak of 24 detected in July of the second year, and remained similar until September (Table 1). The sharp OD increase observed in summer of the second year for SGH and rSP03B was less pronounced for rSP03B+rSP01 (Table 1). Moreover, correlation of antibody response between SGH and rSP03B was stronger (r = 0.77) than between rSP03B+rSP01 and SGH (r = 0.65) (Fig 3). In addition, we detected high correlation between antibodies recognizing rSP03B and antibodies recognizing combination of rSP03B+rSP01 (r = 0.75) (Fig 3).

**Table 1:** Median and range antibody OD values (multiplied by 100) for each used antigen according sampling month.

Study year	Sampling month		SGH	rSP03B	rSP03B+rSP01
		n	Median(range)	Median(range)	Median(range)
First	July	56	5 (3–12)	14 (7–36)	29 (19–49)
	August	53	9 (4–42)*	26 (14–75) *	37 (16–71) *
	September	54	11 (4–56) *	30 (11–165)	42 (19–116)*
	October	34	11 (3–59)	26 (11–93)	40 (17–85)
	December	54	9 (4–50) *	19 (10–81) *	39 (21–100)
Second	January	56	8 (2–49)	19 (1–69)	34 (19–93)
	March	56	10 (4–62)*	22 (9–89)*	38 (11–117)
	July	56	24 (6–161) *	36 (9–223) *	41 (17–177)
	August	56	17 (5–129)	34 (11–154)	41 (18–157)
	September	54	18 (6–194)	31 (11–234)	42 (18–189)
	Total		10 (2–194)	24 (1–234)	37 (11–189)

n = number of dogs

* significant change in the median compared to previous sampling

Within plate repeatability was high for each antigen and the CCC varied from 0.94 (95% CI: 0.93–0.95) to 0.99 (95% CI: 0.98–0.99), depending on the antigen. However, the CCC between plate was moderately high for whole SGH (0.77, 95% CI: 0.73–0.8) and rSP03B (0.88, 95% CI: 0.87–0.9) and low for the combination of rSP03B+ rSP01 (0.53, 95% CI: 0.47–0.59).

### Multivariable relationship between salivary antibodies, season, and *L*. *infantum* infection

Multilevel models confirmed the strong association between antibodies against SGH and recombinant proteins and sampling month, and between SGH and active *L*. *infantum* infection.

**Fig 4:**
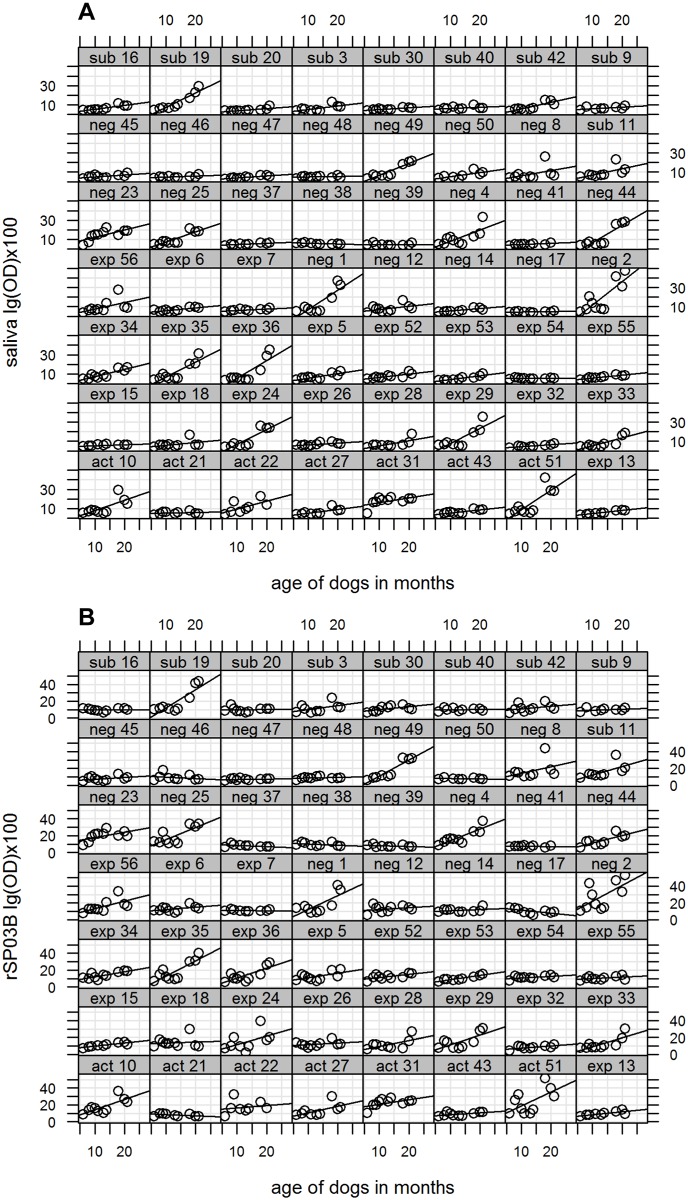
Dynamics of IgG antibodies recognizing SGH (A) and rSP03B protein (B) in individual dogs from CanL endemic locality. *Leishmania* infection status was assigned for 53 dogs according their infection status at the end of the study in March third year and for 3 dogs according their infection status in July second year (dog number 7, 8, 49). OD values were log transformed and multiplied by 100. OD = optical density, neg = negative, exp = exposed, sub = subpatent, act = active.

Tables 2 and 3 present parameters estimates for models including only sampling date (model a) and both sampling date and *L*. *infantum* infection status (model b) for SGH and rSP03B, respectively (for combination of rSP03B+rSP01 data are showed in S1 Table). Estimates for sampling months alone reflect a significant increase in log OD values by September in comparison to July of the first year, when the study started, then they started to decrease in October, and raised again in March and especially in July of the second year, with the highest log OD estimate detected in the following month of September (p<0.05) (Tables 2 and 3, model a). Including dog as a random effect significantly improved the model and the variance estimate indicated that for SGH it was 33% [(10.06/(10.06+20.35))*100] of the variation in log OD dog related. For rSP03B it was 38% [(17.33/(17.33+28.67))*100]. This can be appreciated in Fig 4A and 4B representing the sampling month-specific ODs for the 56 study dogs. Finally, model b shows the sharp increase in log OD between March and July of the second year (Tables 2 and 3) and highlights the strong association between the amount of antibodies against whole saliva and dogs with active *L*. *infantum* infection (p<0.05) (Table 2). The association between active infection and anti-rSP03B antibodies (Table 3) and combination of rSP03B+rSP01did not reach statistical significance. The model also shows that the greatest variation in log ODs between dogs was observed in July of the second year (Tables 2 and 3). There was no evidence of correlation between repeated measures in any of the above models.

**Table 2:** Estimates of the multilevel linear regression model of the relationship between log transformed SGH OD values (multiplied by 100) and sampling time (model a), and *Leishmania* status and sampling time (model b).

Variable	Levels	Estimate	SE	P value
**a) sampling month only**				
Intercept		4.33	0.74	<0.001
**Fixed effects**				
Sampling month	July (first year)	0.00		
	August (first year)	1.56	0.87	0.073
	September (first year)	2.85	0.86	0.001
	October (first year)	2.49	0.99	0.012
	December (first year)	1.60	0.86	0.064
	January (second year)	1.55	0.85	0.069
	March (second year)	2.36	0.85	0.006
	July (second year)	9.21	0.85	<0.001
	August (second year)	7.84	0.85	<0.001
	September (second year)	9.62	0.86	<0.001
**Random effects**	**Variance**			
Dog	10.06			
Residual	20.35			
**b) sampling month and *Leishmania* infection status**	Levels	Estimate	SE	P value
Intercept		4.33	0.11	<0.001
**Fixed effects**				
Sampling month	July (first year)	0.00		
	March (second year)	1.71	0.56	0.003
	July (second year)	8.46	1.2	<0.001
*Leishmania* status	Negative	0.00		
	Exposed	1.39	0.90	0.13
	Subpatent	1.28	2.96	0.67
	Active	5.11	2.38	0.03
**Random effects**	**Variance**			
Dog	0.11			
March (second year)	10.42			
July (second year)	71.52			
Residual	0.51			

SE = standard error

**Table 3:** Estimates of the multilevel linear regression model of the relationship between log transformed rSP03B OD values (multiplied by 100) and sampling time (model a), and *Leishmania* status and sampling time (model b).

Variable	Levels	Estimate	SE	P value
**a) sampling month only**				
Intercept		8.18	0.91	<0.001
**Fixed effects**				
Sampling month	July (first year)	0.00		
	August (first year)	4.15	1.03	<0.001
	September (first year)	5.87	1.02	<0.001
	October (first year)	3.61	1.18	0.002
	December (first year)	2.14	1.02	0.037
	January (second year)	1.53	1.01	0.131
	March (second year)	2.88	1.01	0.005
	July (second year)	10.81	1.01	<0.001
	August (second year)	8.50	1.01	<0.001
	September (second year)	9.67	1.02	<0.001
**Random effects**	**Variance**			
Dog	17.33			
Residual	28.67			
**b) sampling date and *Leishmania* infection status**	Levels	Estimate	SE	P value
Intercept		8.18	0.32	<0.001
**Fixed effects**				
Sampling month	July (first year)	0.00		
	March (second year)	2.26	0.74	0.003
	July (second year)	10.13	1.49	<0.001
*Leishmania* status	Negative	0.00		
	Exposed	1.47	1.11	0.188
	Subpatent	-1.11	3.59	0.758
	Active	4.68	3.14	0.139
**Random effects**	**Variance**			
Dog	2.39			
March (second year)	15.34			
July (second year)	104.54			
Residual	3.31			

SE = standard error

## Discussion

This paper describes for the first time the dynamics and diagnostic potential of antibodies recognizing *P*. *perniciosus* salivary recombinant proteins in dogs following natural exposure to the sand flies over two years.

The study area is considered traditionally endemic for *L*. *infantum* [35]. The rate of dogs with active infection was increasing over the two-year period, and this pattern was in accordance with previous studies carried out in the area [5, 8]. Our results indicate that there is a significant positive association between anti-*P*. *perniciosus* saliva antibodies and active *L*. *infantum* infection. This finding could reflect that these dogs had a greater sand fly challenge compared to other dogs and therefore developed a stronger humoral response against salivary proteins, thus had bigger chance to become infected. Alternatively, because active *L*. *infantum* infection in dogs is characterized by a mixed Th1/Th2 response associated with marked antibody production [reviewed in [36]], it is possible that our *Leishmania* infected dogs were more sensitive to develop a humoral antibody response against sand fly salivary gland proteins. So far, there is only one report describing an association between CanL and positivity for anti-sand fly saliva antibodies. The study, performed in a *L*. *infantum* focus in Apulia region of Italy, showed that *Leishmania* positive and negative dogs did not differ in IgG and IgG1 production against the whole *P*. *perniciosus* salivary lysates but the *Leishmania* positive ones had significantly decreased levels of IgG2 antibodies [12]. However, we did not find any association between antibodies against rSP03B protein and active CanL infection. Although, the use of rSP03B protein as a marker of exposure to sand flies is promising, use of recombinant proteins as risk markers for infection need more investigation.

In the present study *P*. *perniciosus* salivary recombinant proteins, rSP03B (yellow- related protein) and its combination with rSP01 (apyrase) were used. For rSP03B, the repeatability of ELISA test was even higher than for SGH, proving this recombinant protein to be better antigen for large scale studies. The achieved high correlation score between SGH and rSP03B was in agreement with results from previous studies [20, 21]. However, the combination of two recombinant proteins (rSP03B and rSP01) did not show better performance than a single SP03B. Repeated experiments suggest that this could be due to instability of rSP01 protein and its higher susceptibility to repeated thawing and freezing. Previously, better results for recombinant proteins combination were obtained for detection of anti-*Lu*. *longipalpis* antibodies when two recombinant yellow-related proteins were combined together [18].

Kinetics of anti-SGH and anti-rSP03B IgG antibodies developed with similar pattern and were clearly seasonal: rising during summer months when sand fly density is the highest and decreasing during winter months when sand flies are not active. This positive correlation between antibodies against salivary proteins and seasonal abundance of blood feeding insects has been previously reported in mosquitoes [37, 38]. The smaller increase of antibodies against *P*. *perniciosus* salivary proteins during the first transmission season was probably due to the fact that dogs were exposed to the vector for the first time and were moved to an endemic locality in the middle of the transmission season (July). In central and southern peninsular Italy, the sand fly season usually lasts from late May to late October, with two density peaks [25, 39]. The significant increase of IgG antibodies for SGH and rSP03B protein in March compared to January in the second year (Fig 2) remains unexplained, since it cannot be attributed to such an early activity of sand flies. Even in the southernmost region of Italy (Sicily) the earliest collection of *P*. *perniciosus* was recently reported to be in April [40]. The marked increase in antibody levels in the second transmission season is much probably caused by re-exposure of dogs to sand flies following antigenic priming in the previous season. Similar antibody responses to sand fly saliva were observed in mice re-exposed to *P*. *papatasi* [17] and in humans re-exposed to *P*. *argentipes* [11]. Moreover, the vector population density could vary between years due to the different climatic conditions [39] and therefore, the dogs could have been exposed to higher sand fly challenge during the second transmission season. However, as shown by the multilevel models, there was significant variation in the amount of anti-saliva antibodies between dogs, particularly during July of the second year, the reason of which remains unclear. It could be associated with innate differences in their antibody responses to sand fly salivary antigens or in attractiveness to sand fly bites, or with other unknown factors that need to be investigated. Interestingly, it was recently reported that Beagle dogs may exhibit markedly different attractiveness to *P*. *perniciosus* under experimental exposure [41].

In conclusion, the dynamics of antibody response against *P*. *perniciosus* salivary proteins clearly showed seasonal changes due to the expected sand fly abundance. Our study confirmed that recombinant yellow-related protein rSP03B of *P*. *perniciosus* is a valid alternative to whole sand fly saliva as marker of sand fly exposure. Serology tests based on this recombinant protein could be a practical and economically-sound tool for investigations dog exposure to sand flies in endemic settings of zoonotic visceral leishmaniasis.

## Supporting Information

S1 Table Estimates of the multilevel linear regression model of the relationship between log transformed rSP03B+rSP01 OD values (multiplied by 100) and sampling time (model a), and *Leishmania* status and sampling time (model b). (DOCX)
