# Supplementary material for: Canine Antibodies against Salivary Recombinant Proteins of Phlebotomus perniciosus: A Longitudinal Study in an Endemic Focus of Canine Leishmaniasis
Source: PLoS Negl Trop Dis. 2015 Jun 25;9(6):e0003855. doi: 10.1371/journal.pntd.0003855 (PMC4482481; doi:10.1371/journal.pntd.0003855)
Supplement: S1 Table — (DOCX) [file pntd.0003855.s001.docx]

**S1 Table. Estimates of the multilevel linear regression model of the relationship between log transformed rSP03B+rSP01 OD values (multiplied by 100) and sampling time (model a), and *Leishmania* status and sampling time (model b).**

| Variable | Levels | Estimate | SE | P value |
| --- | --- | --- | --- | --- |
| **a) sampling month only** |  |  |  |  |
| Intercept |  | 12.95 | 0.76 | <0.001 |
| **Fixed effects** |  |  |  |  |
| Sampling month | July (first year) | 0.00 |  |  |
|  | August (first year) | 1.74 | 0.87 | 0.045 |
|  | September (first year) | 4.07 | 0.86 | <0.001 |
|  | October (first year) | 2.66 | 0.99 | 0.008 |
|  | December (first year) | 2.51 | 0.86 | 0.004 |
|  | January (second year) | 1.80 | 0.85 | 0.036 |
|  | March (second year) | 2.56 | 0.85 | 0.003 |
|  | July (second year) | 6.31 | 0.85 | <0.001 |
|  | August (second year) | 5.28 | 0.85 | <0.001 |
|  | September (second year) | 6.40 | 0.86 | <0.001 |
|  |  |  |  |  |
| **Random effects** | **Variance** |  |  |  |
| Dog | 11.67 |  |  |  |
| Residual | 20.46 |  |  |  |
|  |  |  |  |  |
| **b) sampling date and *Leishmania* infection status** | Levels | Estimate | SE | P value |
| Intercept |  | 12.95 | 0.30 | <0.001 |
| **Fixed effects** |  |  |  |  |
| Sampling month | July (first year) | 0.00 |  |  |
|  | March (second year) | 2.20 | 0.60 | <0.001 |
|  | July (second year) | 5.85 | 1.26 | <0.001 |
|  |  |  |  |  |
| *Leishmania* status | Negative | 0.00 |  |  |
|  | Exposed | 0.69 | 0.96 | 0.476 |
|  | Subpatent | 3.70 | 3.14 | 0.242 |
|  | Active | 1.97 | 2.50 | 0.433 |
|  |  |  |  |  |
| **Random effects** | **Variance** |  |  |  |
| Dog | 2.51 |  |  |  |
| March (second year) | 8.60 |  |  |  |
| July (second year) | 74.54 |  |  |  |
| Residual | 2.61 |  |  |  |

SE=standard error
